# *Mycobacterium bovis* Infection of Red Fox, France

**DOI:** 10.3201/eid2406.180094

**Published:** 2018-06

**Authors:** Lorraine Michelet, Krystel De Cruz, Sylvie Hénault, Jennifer Tambosco, Céline Richomme, Édouard Réveillaud, Hélène Gares, Jean-Louis Moyen, María Laura Boschiroli

**Affiliations:** ANSES Laboratory Affairs Department, Maisons-Alfort, France (L. Michelet, K. De Cruz, S. Hénault, J. Tambosco, E. Réveillaud, M.L. Boschiroli);; French Agency for Food, Environmental and Occupational Health Safety—Nancy Laboratory for Rabies and Wildlife, Malzéville, France (C. Richomme);; Laboratoire Départemental d'Analyse et de Recherche de Dordogne, Coulounieix Chamiers, France (H. Gares, J.-L. Moyen)

**Keywords:** *Mycobacterium bovis*, red fox, Vulpes vulpes, excretion, bovine tuberculosis, France, bacteria, Tuberculosis and other mycobacterial infections, zoonoses

## Abstract

*Mycobacterium bovis* infection in wild red foxes was found in southern France, where livestock and other wildlife species are infected. Foxes frequently interact with cattle but have been underestimated as a reservoir of *M. bovis*. Our results suggest a possible role of the red fox in the epidemiology of bovine tuberculosis.

*Mycobacterium bovis*, a member of the *Mycobacterium tuberculosis* complex (MTBC), is the main etiologic agent of bovine tuberculosis (TB). In France and other countries in Europe, this ancient zoonotic disease is regarded not just as a problem of cattle but as a concern for multihost communities that include wildlife species such as wild boar (*Sus scrofa*), red deer (*Cervus elaphus*), and badgers (*Meles meles*) (*1*). The red fox (*Vulpes vulpes*) is usually considered a spillover host of TB. Sparse numbers of infected foxes had been found in highly prevalent TB regions, such as Great Britain (*2*). Until recently, the number of infected foxes in TB-endemic zones in France was low (1/68 in Brotonne Forest, Normandy, northwestern France, and 2/61 in Côte d’Or, Burgundy, central-eastern France) (*3*). However, recently an increasing number of reports have described the presence of a substantial number of infected red foxes in Mediterranean habitats in Spain (*4*) and Portugal (*5*), where the disease is highly prevalent in wildlife and livestock. In these cases, the prevalence was high (14% and 26.9%, respectively). Strikingly, most animals did not have TB-like visible lesions in any of these reports.

In a recent study, Payne demonstrated that in Burgundy, France, a region where TB is prevalent in cattle and wildlife, the red fox is the species that most often visits cattle environments (*3*). In 2015, we conducted a small-scale investigation on trapped red foxes (n = 6) in a municipality of Dordogne, a TB-endemic region in southwestern France, where multihost cycles involving cattle, badgers, wild boar, and even red deer and roe deer (*Capreolus capreolus*) exist (*6*). The necropsy examination included detailed macroscopic inspection of lymph nodes and abdominal and thoracic viscera (*6*). We found no TB-like visible lesions; pooled tissue samples (retropharyngeal, tracheobronchial, mediastinal, and mesenteric lymph nodes) were submitted bacterial culture and molecular diagnosis (*6*); urine, feces, and oropharyngeal swabs were taken, and DNA was extracted as previously described (*7*). We performed molecular diagnostic testing using MTBC PCR (IS*6110* and IS*1081*) and the regions of difference and through spoligotyping (*6*), the latter 2 enabling differentiation of the MTBC members when used on highly concentrated DNAs. Of the 6 red foxes, 4 were bacteriology positive; molecular tools enabled identification of *M. bovis* spoligotype SB0120 and MTBC or *M. bovis* excretion in feces of all infected animals (Table). One red fox (RN5) also gave positive results on oropharyngeal swab and urine samples, suggesting *M. bovis* excretion through several routes.

**Table:** Results of direct *Mycobacterium bovis* detection on organs and molecular detection on excretory samples of red foxes, France, 2015*

Fox	Direct detection on organs			Molecular detection on excretory samples		
	Bacteriology	Molecular diagnostics		Oropharyngeal swab	Feces	Urine
RN2	Negative	Negative		Negative	Negative	Negative
RN3	Negative	Negative		Negative	Negative	No sample
RN4	*M. bovis* SB0120	MTBC		Negative	MTBC	No sample
RN5	*M. bovis* SB0120	MTBC		MTBC	MTBC	*M. bovis* SB0120
RN6	*M. bovis* SB0120	*M. bovis* SB0120		Negative	MTBC	Negative
RN7	*M. bovis* SB0120	Negative		Negative	*M. bovis* (partial spoligotype profile)	Negative

*MTBC, *Mycobacterium tuberculosis* complex.

Although molecular detection does not prove that bacilli are viable, this method is particularly useful for monitoring shedding in environmental samples—especially fecal material—where bacterial culture has poor sensitivity. Molecular quantification on these samples seems to correlate strongly with the animal’s infection level (*7*).

Fecal excretion may result from digestive infection (*8*). Infection in mesenteric lymph nodes, observed in other carnivores, suggests that the primary route of transmission of infection is through the digestive tract and strongly suggests fecal excretion (*5*). Fox RN5 may be considered a super-shedder or super-excretor (*9*) because shedding of the tuberculous bacillus by several routes was observed (e.g., oropharyngeal swab samples, feces, and urine). Super-shedders have been described as responsible for a disproportionately large amount of *M. bovis* excretion from the infected animal with a substantial role in the transmission and maintenance of TB in multihost pathogen systems (*9*), raising the question of the role of the fox in the epidemiology of TB.

Infection in foxes has been suggested to result from scavenging on infected wild ungulate carcasses (*4*). Alternatively, because foxes may inhabit disused badger setts and vacant parts of occupied setts, they could acquire infection from the contaminated environment (*10*). This possibility may constitute a risk regarding TB because the fox has been described as the most frequently observed species directly interacting with cattle and visiting farm facilities (*3*). 

The presence of visible lesions alone does not appear to be a good indicator of *M. bovis* infection in carnivores such as foxes, which often lack macroscopic lesions. The nature of *M. bovis* infection and the host response are likely to vary widely among species, making simple generalizations about pathology difficult to determine (*2*). The prevalence of kidney lesions has been reported to be low in wildlife species, which may be explained by the difficulty of detecting TB lesions in organs with a large parenchyma or the presence of microscopic lesions often missed by gross pathology (*3*,*8*). Further studies are needed to investigate urinary excretion and the prevalence of kidney TB lesions in red fox and in wildlife in general. Together with this study’s results, these observations highlight that the role of red fox in the epidemiology of TB needs further investigation.
